# A microcosting approach for planning and implementing community-based mental health prevention programs: what does it cost?

**DOI:** 10.1186/s13561-024-00510-w

**Published:** 2024-05-21

**Authors:** Sharmily Roy, Henry Shelton Brown, Lisa Sanger Blinn, Sarah Carter Narendorf, Jane E. Hamilton

**Affiliations:** 1 School of Public Health, University of Texas Health Science Center, Houston, 1200 Pressler St, Houston, TX 77030 USA; 2 The Harris Center for Mental Health and IDD, 9401 Southwest Freeway, Houston, TX 77074 USA; 3 Graduate College of Social Work, University of Houston, 3511 Cullen Blvd, Houston, TX 77204 USA; 4 Department of Psychiatry and Behavioral Sciences, McGovern Medical School, University of Texas Health Science Center, Houston, 1941 East Road, Houston, TX 77054 USA

**Keywords:** Community based behavioral health program, Mental health promotion, Preventive/preventative program planning, Microcosting, Activity-based costing, Start-up costs, Implementation costing

## Abstract

**Background:**

Estimating program costs when planning community-based mental health programs can be burdensome. Our aim was to retrospectively document the cost for the first year of planning and implementing Healthy Minds Healthy Communities (HMHC), a mental health promotion and prevention multi-level intervention initiative. This Program is among the first to use the Community Initiated Care (CIC) model in the US and is aimed at building community resilience and the capacity for communities to provide mental health support, particularly among those disproportionately impacted by COVID-19. Our objective is to share our methods for costing a program targeting 10 zip codes that are ethnically and linguistically diverse and provide an example for estimating the cost of a mental health prevention and promotion programs consisting of multiple evidence-based interventions.

**Methods:**

We used a semi-structured interview process to collect cost data through the first year of program planning, start-up and initial implementation from key staff. We calculated costs for each activity, grouped them by major project categories, and identified the cost drivers of each category. We further validated cost estimates through extensive literature review. The cost analysis was done from the provider’s perspective, which included the implementing agency and its community partners. We delineated costs that were in-kind contributions to the program by other agency, and community partners. Sensitivity analyses were conducted to estimate uncertainty around parameters.

**Results:**

For the first year of the development and implementation of the program, (funded through program and in-kind) is estimated at $1,382,669 (2022 US$). The costs for the three main activity domains for this project are: project management $135,822, community engagement $364,216 and design and execution $756,934. Overall, the cost drivers for the first year of this intervention were: hiring and onboarding staff, in-person community building/learning sessions, communications and marketing, and intervention delivery.

**Conclusion:**

Implementation of community-based mental health promotion and prevention programs, when utilizing a participatory approach, requires a significant amount of upfront investment in program planning and development. A large proportion of this investment tends to be human capital input. Developing partnerships is a successful strategy for defraying costs.

**Supplementary Information:**

The online version contains supplementary material available at 10.1186/s13561-024-00510-w.

## Introduction

### Background

When considering whether to implement community-based mental health promotion and prevention (MHPP) programs, cost estimation is essential for determining program affordability and advocating for program funding [1–5]. Stakeholder-driven, community-based MHPP programs are posited to provide cost-effectiveness [6]. However, compared to community-based mental health treatment programs, resources allocated to prevention programs in the US are very low in terms of number of community-based programs and funding per program [7–9]. In particular, cost studies for community based participatory approaches are limited [10, 11]. As a result, there is a dearth of economic analyses in this area, which is a barrier for uptake of evidence-based preventive health practices [6, 12–14]. Even when there is available research, it is often difficult for non-economists to decipher relevant meaning for their settings, hampering planning and cost estimation [15–17]. Cost estimation, as contrasted with cost-effectiveness analysis, which depends on ex-post outcomes, is rarely an academic subject unto itself, but cost estimation is what is needed in the planning stages [14, 17, 18]. Accordingly, information on how to estimate costs for community-based interventions during earlier stages of program development and planning, prior to implementation, is a critical gap that needs to be elucidated for planners, administrators and policy makers [19–21].

This study analyzes the first phase of planning and development costs of a 5-year, federally funded stakeholder-driven, community-based MHPP program, *Healthy Minds, Healthy Communities (HMHC)* in Harris County, Texas, launched in 2021. The Program is funded through the American Rescue Plan Act of 2021 (ARPA) funds to ameliorate mental health burden due to COVID-19. HMHC was implemented by a local mental health authority (LMHA) in 10 zip-codes with population range of 23,000–71,000 with a total estimated population of 271,940 across these 10 zip codes of which approximately 190,000 belong to a racial or ethnic minority group, and where 44% of residents speak a language other than English at home [22, 23]. The county received $8.93m from the ARPA Stimulus bill for community based preventive mental health intervention aimed at mental wellness and resiliency. Key outcomes include increased knowledge of national suicide prevention hotline number (988) implemented in the US in 2022, reduction in suicide risk, reduction in firearm suicide specifically, and improved mental health resilience skills in the key populations in the targeted zip-codes.

During the planning phase, the LMHA identified 9 zip codes for project implementation based on identified health disparities including underutilization of community-based mental health services, disproportionate impact by COVID-19, and a greater number of suicide deaths in 2020. One additional zip code was specifically included due to high rates of firearm suicide. There have been a few microcosting studies in MHPP that are relevant to planning and implementation of a varied mix of interventions in multiple sites serving different demographic mix and needs. Microcosting studies have primarily focused on single-site implementation [19, 24]. Very few community based MHPP studies are found in the comprehensive Tufts registry of cost-effectiveness studies [25]. However, identifying and accurately costing key cost-drivers improves the quality and value of microcosting studies to provide evidence for planning and implementation decisions [26]. Hence, the current analysis was conducted as a first step to support further economic analysis in microcosting estimation in mental health promotion and prevention [26].

## Methods

### Design

This analysis utilizes the following data sources: the use of ARPA funds; agency personnel time and other administrative resources; and county government’s and community-based organizations’ support and resources, to examine cost of developing the HMHC program. Similar to a cost study of a state government implemented behavioral health program, we report the value of costs of services, expertise and other resources, regardless of whether any money was exchanged [27].

This retrospective microcosting analysis incorporated all direct and indirect costs including overhead, such as in-kind donated space from the county, and personnel time from the implementing agency, that was not funded by the program budget. A series of semi-structured interviews (*n* = 4) with HMHC project leaders (project director and project manager) with a standardized interview instrument to collect information for the microcosting study was administered (Table 7 in Appendix). The interview information was used to develop the standard tables (Tables 1, 2, 3, 4 and 5). Additional data sources included email calendars, payroll data, invoices, purchase orders, public records data, subscription payments, and contracts. The information gathered and consolidated went through validation with program managers.

**Table 1:** HMHC cost summaries

	**Personnel Hours**		**Total Personnel Costs**	**Other Costs**	**Grant Funded Costs**	**In-kind Costs**	**Total Costs**	**Total cost per capita (Cost/potential beneficiary)**
Category	**Lead**	**Others**						
Project Management	1,120–1240	52	**$107857**	**$27965**	**$135,822**		**$135,822**	**$1.06**
Community Engagement	233	4932	**$200,435**	**$79,580**	**$269,089**	**$84,201**	**$364,216**	**$2.78**
Design and Execution (internal)	132	2440	**$7142**			**$225,000**^**a**^	**$539,136**	**$1.88**
Communication Contracts (D&E external)				**$524,792**			**$524,792**	**$4.00**
Overhead (10% of total)							**$125,697**	**$.97**
Total Cost			**$315,434**	**$632,337**	**$936,905**	**$309,201**	**$1,382,669**	**$9.72**

^a^Material support of $225,000 includes personnel time of 2400, and is estimated at the market price charged to other agencies for this service

**Table 2:** Domain costs

		**Personnel Hours**^**c**^		**Total Personnel costs**^**c,d**^	**Other Costs**^**b,c,d**^	**Grant Funded Costs**	**In-kind**^**c**^		**Total costs**	**Total cost per capita (Cost/pop)**
**Category**	**Activity**	**Lead**	**Others**				**Personnel**	**Material Support**		
**Project Management**	Hire program staff^a^ (63% of domain cost; 7% of total cost)	360–480		$56,193-$74,924	$19565	$56193-$74924.15			$75758.11-$94489.15 (Mean:$65559)	$ 0.66
	Project metrics development	20		$1,050		$1050.59			$1050.59	0.001
	Staff Development	260	52	$15,641	$8400	$24041			$24,041	$0.19
	Marketing RFP Development	480		25606		$25606			$25,606	$0.20
**Total Project Management Cost**				**107857**	**27965**	**135822**			**135822**	**$1.06**
**Community Engagement**	Precinct mtgs	90		$4,728		$4728	$7,780		$12,508	$0.10
	Stakeholder identification	110		$5,778		$5778			$5778	$0.04
	Virtual Launch				$420	$420			$420	$0.002
	Community Learning Circles (CLC)	33	132	$6770	$69195-$71745 (Avg: $70,470)	$75965-$78515 (Avg: $77,240)	$70421	$6000	$152386-$154936 (Avg:$153,661)	$1.15
	Community prioritization (53% of domain; 15% of total)		4800	$183,159	$8,690	$191,849			$191,849	$1.49
**Total Community Engagement Cost**				**$200,435**	**$79,580**	**$269,089**	**$84,201**		**$364,216**	$2.78
**Design and Execution**	Comm and outreach plan	60		$3122	79	$3122			$3122	$0.02
	Communication contracts^a^ (68% of domain; 41% of total)				$515625	$515625			$515625	$4.00
	My Strength app									
	Capacity for ASK (T4T)	24	40	$1554	$5729	$7283			$7283	$0.06
	Identify culturally appropriate program	48		$2466	$3,437	$5903			$5903	$0.05
	Mental Health First Aid training		2400				$225000		$225000	$1.75
**Total Design and Execution Cost**				**$7142**	**$524,792**	**$531,994**	**$225,000**		**$756,934**	$5.88
**Total Cost across Domains**				**$315,434**	**$632,337**	**$936,905**	**$309,201**		**$1,256,972**	$**9.72**

^a^Denotes cost driver

^b^Actual cost

^c^Estimates from program. MHFA training time is 2400h. This training costs $1500 per session when contracted by various schools, non-profits, and other agencies. ~ 50 sessions were accessible in the target community

^d^Estimates from literature

**Table 3:** HMHC lead personnel

Position	Start Date	Annual Salary	Effort in Calendar Months
Project Outcome Lead	11/16/2021	$76,314	12
Program Manager	02/15/2022	$72,954	6
Communications Lead	10/15/2021	$77,500	12

**Table 4:** HMHC other personnel

Position	Start Date or Duration	Annual Salary	Effort in Calendar Months
Coordinator	01/18/2022	$53,601	12
Community Engagement Coordinator	05/24/2022	$60,275	6
Community Engagement Specialist	09/13/2022	$57,100	3
Community Engagement Associate	09/26/2022	$52,601	2
Relief Program Assistant	05/10/2022–08/30/2022	$20/hour	3
Contract Program Assistant	9/1/2022	$40/hour	1
Community Engagement Coordinator	01/18/2022–04/26/2022	$53,601	3

**Table 5:** Non-personnel time and skills contribution by community stakeholders

Position	Type of Skills Support (BLS)	Time/Effort in Calendar Months	Start and End Dates	Estimated Cost of Contribution (BLS)
Program & Outreach Manager	21–1099	.25	04/15/2022-12/31/2022	Sal: $75,604 per public records
Director, Health Care & Social Services	21–1019	.25	04/15/2022–12/31/2022	Sal: $103,564 per public records
Pastor	21–2011	.25	05/01/2022-12/31/2022	Avg. mean annual wage: $57,230
Community Engagement Coordinator	21–1099	.25	05/01/2022-12/31/2022	Sal: $61,887 per public records
Community Engagement Coordinator	21–1099	.25	05/01/2022-12/31/2022	Sal: $75,959 per public records

**Fig. 1:**
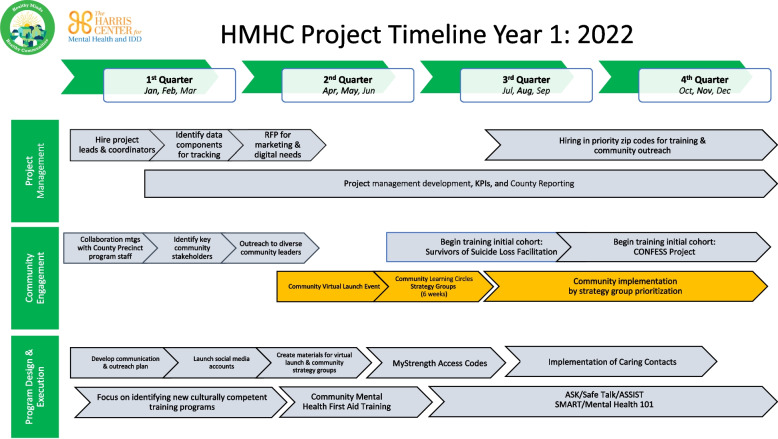
Project implementation timeline year 1

During the interviews, program leads provided information collected retrospectively from calendars, meeting minutes and attendance logs to estimate personnel hours and activity details. The research team collected information on the involvement of all individuals in each of the major activity categories (Fig. 1), role and the specific nature of the work in narrative style. This included details of who conducted the activities, the date of activities, time spent on the activity and a description of the activities. Estimates of time cost of partners, county personnel and stakeholders were developed utilizing the Bureau of Labor Statistics (BLS) occupational profiles, and county median salary information for associated pay grades and other publicly available data [28]. The estimate of the effort time is based on project leaders’ description of activity and duration in each of the categories of activities. In some cases, an estimated range is reported, while in others, a closer estimate is utilized. The implementing agency provided salary and fringe benefit information, and these are included in personnel cost. All costs are provided in 2022 USD. Staff time per hour rates were estimated by averaging actual salary and pay information. An estimate of 1920 annual hours (i.e., 10 holidays, estimated two weeks of sick days and two weeks’ vacation per FTE) is used for personnel hours for this analysis, similar to that identified in literature of annualized work time measure conducted by state government employees [27]. The activity estimates include travel cost, based on IRS reimbursement rate, applied to state and local agencies which ranged from $0.58/mile to $0.62/mile in 2022 [29]. The employee fringe rate is 32.16% at this institution. Cost data can be delineated into three categories of precision: 1) actual cost (i.e., invoices, program staff salary/fringe documents, county space rental costs), 2) estimates from program (i.e. labor hours per activity, county salary grade range), and 3) estimates from literature (aggregated annual hours and proportion of overhead costs) [27].

### Setting

The Program is a multi-year, multi-level intervention initiative aiming to promote mental health and prevent mental illness. Interventions are tailored in each zip code and include group sessions led by trained facilitators promoting mental health and resiliency through outreach at community fairs and other locations. Strategies include dissemination of mental health stigma reduction messages by social media influencers, promoting a suicide prevention hotline (988), and partnering with key community based organizations serving the target populations to support the implementation of mental health resilience into existing programming. Additionally, training in evidence-based interventions, Mental Health First Aid and ASK, are provided in the community to increase suicide prevention skills and provide peer support and initial triage for mental health needs [30, 31].

The Program draws on the Community Initiated Care (CIC) model and is adapted based on significant input from local stakeholders [32]. CIC is a model that aims to increase the capacity of communities to engage in mental health prevention by “task-sharing” or “task-shifting” with trained community members instead of relying solely on limited formal services from licensed mental health professionals [33–39]. Task sharing or task shifting in mental health refers to basic mental health service being provided by trained community members, who may not have years of education and degrees of a traditional mental health specialist. These tasks can include screening for signs and symptoms, active listening, and triage to connect with mental health services [37, 38]. This model is a response to the shortage of behavioral health providers and its disproportionate effect on historically underserved communities [40, 41].

The period of this study covers the first year of program development and implementation (2022). Program development within the HMHC initiative consisted of preliminary planning, such as choosing the communities where the program would be implemented, identifying community needs, and finding ways to respond to the needs. Costs in this area included an iterative process of community consensus building. Implementation of effective programs requires a quality improvement approach – where lessons learned while doing, are implemented rapidly into program activities [42]. Various levels of county and agency leadership, along with federal guidelines and parameters for use of funds, played a role in program development, and costs. Planning costs are typically not included in cost-effectiveness for philosophical reasons but are certainly relevant in the planning stage of a program.

#### Costs perspective

The cost-analysis was conducted from the provider perspective, based on information obtained from the LMHA, which is the implementing and host agency of the HMHC program. Careful planning and timely investments are important to program success from the provider’s perspective [43]. This perspective was chosen to guide microcosting methods [43, 44].

Costs incurred prior to service delivery, including grant application, proposal development and program planning costs is also available (Appendix 2). Key staff and initial program implementation costs remained the same regardless of the scale and number of communities where the interventions were implemented and community member participation. Thus, once set up, these costs did not increase as the project scaled up. Additional costs, such as stakeholder engagements, number of different evidence-based interventions implemented, and other staff increased with the quantity of community participation, such as facilitation of group sessions, and World Café style community listening sessions called *Community Learning Circles* (CLCs) [45]. For this study, we define in-kind costs as any support outside of normal job duties by anyone in the LMHA or county government. These in-kind contributions enabled cost-savings and carry forward of limited grant funds. With COVID-19 mitigation and work from home protocols, there was little shared facility and utilities in day-to-day operations. Overhead administrative services are calculated as 10% of direct costs and notated in program summary (Table 1).

### Measures

#### In-kind personnel and material support

This program received substantial resource support from the LMHA (implementing agency), community partners, and county government. Time and effort contributed to the program which are in-kind and not funded through the program are delineated (Table 5). The title, level/grade, activity, salary and calculated effort during the 12-month period covered in this study was collected. Some partnering organizations utilize a fiscal year and others, calendar year. Therefore, some costs that span a 15-month period are prorated to a 12-month calendar year of January-December 2022. Provision of services in community settings require different infrastructure and resources than in clinical settings [46]. Since this was the first time the LMHA implemented a community-based program, most costs do not have joint objectives and are associated principally for the implementation of this program. In-kind support was given in specific areas: Mental Health First Aid training, community engagement consultation, and donation of meeting spaces and is notated whether this support is through personnel time or materials including physical infrastructure and supplies.

#### Program cost description

Activity-based cost or microcosting approach was used in this analysis. As much as possible, the details on quantities (hours of labor) and prices are provided separately to allow other researchers to adjust these components that would result in a better fit with the local context [37]. The personnel hours involved for leads, and other staff, inputs such as materials and services are shown. This includes services and goods where there was a monetary charge to the program budget, and those which represented in-kind contributions from the host agency or other community collaborators. Costs whether paid for by program funds or estimated from in-kind and personnel support are included in cost estimates for the program. The cost driver for the activity is also identified and demarcated (Table 2).

The program segmented the activities for project management purposes (Fig. 1) into the following three core domains: 1) Project Management, 2) Community Engagement, and 3) Program Design and Execution. These domains are used to map the costing information and analysis.

#### Outcomes

The multi-level intervention initiative is aimed at various at-risk populations for suicide, isolation and other mental health sequalae across each zip code who are likely to benefit from the various interventions planned as part of the HMHC program. Program interventions target aging, school age youth and minority populations in the zip code (which account for a greater share of the mental health burden), as well as behaviors such as safe gun storage among gun owners. Minority population in the US, as defined by Centers for Disease Control and Prevention (CDC) includes American Indians (including Alaska Natives, Eskimos, and Aleuts); Asian Americans; Native Hawaiians and other Pacific Islanders; Blacks; and Hispanics. In order to derive the target population, the minority population in each zip code was calculated from the American Community Survey 2020, 5-year estimates for each zip code [22]. Weights based on proportions in each community were utilized to derive youth, elderly and minority populations across the zip-codes in order to ensure the number of people likely to receive the interventions were not double counted. These groups may however be exposed to the multiple interventions which are part of the program, at different times, and different level (e.g., schools, CLCs,).

### Sensitivity analysis

Two-way sensitivity analysis was applied (Table 6). First. the cost was explored by varying total cost of each activity, including all inputs and components by 10% which may give an indication of the cost of implementing the activities in different markets around the US, with varying costs of goods and services. The second level in the sensitivity analysis varied the labor hours by 10% for all activities for both the leads and other staff. For instance, when hiring staff, the total months needed to hire and onboard 3–4 months was not varied, instead the cost was explored by varying the amount of time effort of 25% utilized for the initial analysis by 10% (15% and 35%) during the same period.

**Table 6:** Sensitivity analysis – varying total costs and personnel time

Activity	Total Cost	-10% Total Costs	+ 10 Total Costs	Range	Total Personnel Costs	(-10% time)	(+ 10% time)	Range (time change)
Hire project staff	$85,124	$76,611	$93,636	$17,025	$65,559	$50,573	$79,841	$29,268
Identify data	$1,051	$946	$1156	$210	$1,051	$946	1,156	210
Staff development	$24,042	$21638	$26446	$4,808	$15,642	$14,078	17207	$3128
Marketing RFP development	$25,606	$23,045	$28,167	$5,121	$25,606	$23,045	28,167	$5121
Precinct mtgs	$12,508	$11,257	$13,758	$27,164	$4,728	$4255	5200	$946
Stakeholder identification	$5,778	$5,200	$6,356	$1,156	$5,778	$5,200	6356	$1,156
Virtual launch	$420	$378	$462	$84	NA	NA	NA	No Change
Learning circle	$14,7710	$132,939	$162,481	$29,542	$6,770	$6,093	7448	$1,354
Community prioritization	$191,849	$172,664	$211,034	$38,370	$183,159	$16,4843	$201475	$36,632
Develop comm and outreach plan	$3,122	$2810	$3,434	$624	$3,122	$2,809	3,434	$624.37
Communications Contracts	$515,625	$464,062	$567186	$103,125	NA	NA	NA	No Change
MyStrenght app					NA	NA	NA	No Change
Identify culturally appropriate prgm	$5,903	$5,313	$6,494	$1181	$2,466	2219	2712	$493
Mental Health First Aid training	$225,000	$202,500	$247,500	$45,000	NA	NA	NA	No Change
T4T-ASK	$7,283	$6,555	$8,012	$1,457	$1,554	1399	1709	$311

## Results

For the first year of the Program, the cost (funded through program budget and in-kind) was estimated to total $1,382,669 (2022 US$). We conducted microcosting to calculate the cost within each domain and identified the cost drivers of each domain. The number of people served in the population is not recorded in the first year of early implementation, We also derived a per capita cost for the community-based intervention activities based on estimated numbers of the target population of the initiative (minority, elderly, school age children) and estimated beneficiaries of the Program to be 128,695 across the zip codes. Overall the cost drivers for the first year of this intervention were: hiring of staff (63% of cost within domain;7% of total program cost; $0.66 per capita cost), community prioritization or personnel time in field outreach to identify and develop relationships with key informants and leaders (54% of domain cost;15% of total program cost; $1.49 per capita cost), communication contracts with two firms (41% of domain costs; 68% of total program cost; $4 per capita cost). In-kind contributions represent provider cost from the implementing agency and its partners but were not part of the program budget. These were delivery of Mental Health First Aid—an evidence-based intervention (EBI), over a year; and partner contribution to *CLCs* The following provides details of each domain and what is contained in each activity area’s ingredients.

### Project management

Microcosting data begins with the domain of internal project management. Under the internal project management umbrella, there are three program key personnel who serve as project leads: (1) Project outcome lead, (2) Program manager, and (3) Communications Lead. The salaries for each position range from $72,954 to $77,500 (Table 3). Hiring project staff included two key ingredients: Staff time during search and onboarding, and IT equipment and supplies. The personnel cost of this activity (wage and fringe) is about $56,193 to $74,924 (Table 2) and are estimates from program staff regarding their staff time effort. The Program created parallel job descriptions to allow diverse applicants to be eligible. The three leads estimated they spent 25% of their time for 3–4 months in the hiring process. The Program made a substantial investment in creating job descriptions and recruiting and onboarding staff. This consisted of activities such as developing appropriate job descriptions, scanning applications, skills identification, and interviewing. This was the first implementation of community-based staff for the agency and required a change to protocols. Efforts were made towards parallel criteria (i.e., years of experience in lieu of degree, etc.,) that would attract candidates from the communities served.

Additional activities included 20h setting up project management metrics representing a cost of $1,050. The Project outcomes lead spent an estimated 260h on staff development planning and other staff spent about 52h (1 h a week) attending the activity events. Staff development activity focused on topics such as implicit bias, attitude and self-awareness. Staff development costs included food, booklets and other resources for training ($100/month, 7staff, 12 months) resulting in personnel cost of about $15,641 and $8,400 in other costs.

Like the staff hiring process, considerable time was spent building up capacity through contract mechanisms. The digital media lead spent about 50% effort during Q1 and Q2 developing descriptions and needs through request for proposals (RFPs) and selection of contractors for activities such as outreach, marketing and other communications activities.

Primary cost drivers in this domain were 1) hiring Program staff, followed by 2) development and awarding RFPs for community activities. This domain’s cost driver, hiring Program staff represents about 60% of the domain’s cost and 7% of the total costs.

### Community engagement

The program staff identified key stakeholders, developed tailored communication plans for their target communities, and conducted face to face group sessions (CLC) and 1–1 sessions with community leaders (Community prioritization). Several rounds of stakeholder meetings took place, and 90 h (cost: $4,728) of program lead’s time is estimated for these. An estimated 10 h per community, representing 100 h was spent identifying and inviting engagement of stakeholders, and approximately an hour is estimated specifically for outreach and awareness to key leaders in each community (110 total hours; cost: $5,778) was spent by Program staff. Also estimated in-kind costs of the county staff time supporting the relationship building is estimated to total $7780.

The virtual launch consisted of communication and social media presence. Some of the communication and media consisted of extra add-on services for Zoom meetings ($150/year), MailChimp ($30/month starting in March 2022), and targeted ad buys on Facebook and Instagram took place for the zip codes. The cost of social media and other services came to $420.

In-person launch of the Program in each of the 10 zip codes, was represented through CLC. The space was donated/in-kind, which has a value of over $6,000, and took place in church and community centers, facilitated by the respective county commissioners’ offices, and other community leaders. Stakeholders contributed to facilitating efforts of the project staff and leveraging buy-in, essential to success in the target zip codes. This monetized staff time for precinct staff and community leaders is estimated to total more than $70,000 (Table 5). Event materials include: food at each event (totaling $3195–3745), promo items and giveaways (estimated cost $10,100), and brochures and flyers (estimated cost range $6900–8900). The CLCs also include a visual graphic recorder ($49,000), who facilitated appreciative inquiry in a world café style for each CLC [40]. All included, the activity of the CLCs, the first in-person event of the project is estimated to cost between $152,386 to $154,936, with ARPA funds attributed to half ($75,965- $78,595) of the inputs and another half coming from the county and community in-kind ($76,421), at no cost to the program budget.

The Community Prioritization activity consisted of community canvassing. In the target zip codes, the staff spend an average of 20 h per week identifying and building relationships with key members of the community. Identifying key community stakeholders, influencers and leaders, to support dissemination and buy-in is about 50% of the workload for the non-lead staff. This is about 4,800 personnel hours. Personnel cost for this activity was $183,159. The teams engaged the broader community by participating and tabling at area events and festivals. These events have included health fairs, county kickoff of new buildings, or resource fair for back to schools, trick or treats, and vaccination clinics. The materials purchased for these have included carrying carts/go boxes, tents and tables, and materials including staff uniforms estimated at $8,690. The total cost of community prioritization was $191,849.

Primary cost drivers in this domain were 1) planning and convening CLCs and 2) Community prioritization. Both also include upfront costs for materials and services for use beyond the activity. Community prioritization represents, as the main cost driver is 53% of domain costs and 15% of total. This domain includes investments in fixed cost items, including marketing materials (e.g., tents, brochures and banners) that can be used for the other activities and future project years. Community prioritization was an important investment in building community and trust across the 10 zip codes.

### Program design and execution

Activities from the timeline (Fig. 1) were conducted as part of program design and execution included communication, planning and implementation, internal efforts at communication and planning for the target communities. This represents personnel cost of $3,122. The project contracted services to two communications and marketing firms, for digital marketing, web design, and other activities. The two activity areas: creating materials for virtual launch and the launch of social media, as described in the project management activities are attributed to these two firms. The firms each received $257,813 to carry out deliverables, through August 2023. These contracts totaling $515,625 represent the main cost driver in this domain and for the first-year costs (68% of domain; 41% of total). Program staff capacity has been raised through training 4 trainer (T4T) efforts with all 8 staff attending *AS* + *K? About Suicide to Save a Life* training where available. Each training course is assumed to be a full workday representing 24 personnel hours for three program leads, and 40 of the other staff representing $1554. These training courses took place in other parts of the state, incurring travel and lodging costs. T4T training resulted in staff building competence to conduct ASK training, when it is implemented in future program years. The cost of this activity totaled $7,283.

The implementing agency, the LMHA conducted Mental Health First Aid training about three times a week in the communities where HMHC program is targeted, and they have an average attendance of 30 people. The fee for implementing this program is usually $1500 per session, which over 50 weeks represents an in-kind contribution of service. With two facilitators, we also estimate this as contribution of 2400 h of personnel time from the community training team at Harris Center, the implementing agency (this time is not part of the HMHC project budget).

Two staff leads attended a national conference to learn more about evidence based mental health programs aimed at Latino communities and identify culturally appropriate programs. The cost of attending this event was $3,437, with lodging, registration and per diem. The total cost for two program leads, where we attributed three 8-h workdays, in addition to conference fees, travel and lodging was $5,903.

The HMHC program conducts Mental Health First Aid training about three times a week, and they have an average attendance of 30 people. The fee for implementing this program is usually $1500 per session, which was estimated to be conducted over 50 weeks and represents an in-kind contribution of service by the implementing agency. With two facilitators, this contribution is estimated as 2400 h of personnel time from the community training team at the implementing agency. This contribution from the implementing agency is valued at $225,000 and not attributed to the ARPA funding received for this program.

### Sensitivity analysis

Program development requires a high level of human capital where costs are inherently uncertain due to the iterative process. In the two-way sensitivity analysis to explore areas of uncertainty facing future implementers, the more labor-intensive activities of hiring project staff and community outreach in the field (community prioritization) had the most variation in costs.

## Discussion

Program development is akin to development costs for new health technologies [14]. There was an opportunity cost to forgoing other types of programs and focusing on community-based MHPP and implementing the CIC model. The scope of this project is larger than most similar projects being implemented for the first time, which are often pilot project implementations, with fewer interventions. The scale of this project is also larger – implementation in all 10 zip codes at once–and tailored to the needs of each zip code with large variation in the community profile. Community partners helped the program leaders learn more about key events and advised on where engagement would be most beneficial. The knowledge of these key informants in community relationship building was an important investment towards future activities. This type of pandemic related disaster funding may be more common as climate related and other types of disasters affect communities, where resiliency building is a necessary personal and community need. The communities in the zip codes included have experienced the cumulative impact of multiple environmental disasters in the last decade [47]. Through a community-based process, the HMHC team identified the need for a broader focus on resiliency across multiple catastrophic events rather than a narrower focus on suicide, as initially intended [35, 45, 47]. In a microcosting study of intervention development, Lairson and colleagues found personnel contributing 69% of the cost [48]. This study found similar results, in personnel being the highest cost activity for program development in the first year of implementation. A considerable amount of in-kind contributions was possible through engagement and the strength of the agency to leverage relationships to engage county and community stakeholders. The program received substantial support from the implementing agency and community. At the same time, the program represented a change in mission to health promotion and prevention for the implementing agency, a LMHA, which diverged from the normal operations of the organization which traditionally focused on direct clinical services to the seriously mentally ill population.

Various conceptualizations of intervention development or program planning share the notion of phases or stages of development or refinement. Information is an economic commodity which has a production cost and value [49]. There is a time cost in testing a program for fit in a community and opportunity cost in forgoing other alternatives. Although not shared in cost tables, the program staff identified and explored steps to implement several evidence-based activities, which after assessing community dynamics, were deemed to not be a good fit, or hard to implement in the first year. Costs are not attributed to planning and exploratory activities related to these evidence-based interventions. The program implemented and was most successful is delivering *Mental Health First Aid*, an evidence-based program, aimed at developing mental health resilience in diverse settings [30]. This was followed by ASK training capacity building among staff, with the intention of intervention delivery taking place in the next year [50]. By leveraging community partners’ strengths and assets, the Program was able to conserve funds during the planning stage, accomplishing more with less funds than originally planned. Hence, more funds will be available for the remainder of the project during program implementation and delivery.

This exercise serves as the basis for structured cost data collection which will serve as templates for recording program details and costs prospectively for future years of this program, aid in cost effectiveness analysis. Utilizing this information can support planning for others planning similar types of community-based prevention programs. Sensitivity analysis information may give an indication of the cost of implementing the activities in different markets around the US, with varying costs of goods and services. The learning curve and time effort might vary in implementing similar activities. Additionally, many of the efforts that required extra time due to COVID-19 risk mitigation practices may not be present for future implementers.

The precision in data collection and burden and acceptability of the tools in the workflow process was a challenge. Orienting organization members on use of costing tools can be a resource intensive exercise [46].

## Conclusions

The start-up costs of this community based MHPP program can inform future program planners, implementers and funders. The CIC model follows a community participatory approach and is a labor-intensive process. This paper describes the upfront costs related to the real-world application of the model. The model includes principles and approaches to community engagement, which can be applied to adaptation and implementation of evidence-based interventions and program development in behavior health. Cost methods utilized in this study provide the level and detail of information that can help implementers understand the monetary value of program planning and development. This microcosting study also shows estimates of monetary values of community partners’ contributions which can help planners who may have a different mix leveraged in kind support. This level of detail shows highly visible activities, such as out-of-town workshop attendance by staff, might have a small impact on budgets. Additionally, for those implementers of behavioral health programs and services considering upstream promotion and prevention interventions, an area where there is limited cost information, the activity-based costing analysis can aid in program design decisions and budget planning. While this first year of program planning is limited in terms of outcomes and outputs, the program cost information can be used universally by others in their first stages of implementation. With more tools, implementers may be more likely to venture into these areas of behavioral health promotion and prevention programming.

### Supplementary Information

Supplementary Material 1.

## Data Availability

The datasets used and/or analyzed during the current study are available from the corresponding author upon reasonable request.

## Appendix

**Table 7:** Contributions from Harris Center Staff

Position	Annual Salary	Effort in Calendar Months
Executive Leadership	Range: 135,000 – 167,000	2
Program Manager	Range: $70,000 – 90,000	2
Senior Director	Range: $90,000 – 120,000	1
Government Affairs Director	Range: $80,000 – 115,000	1
Business Manager	Range: $70,000 – 90,000	.5
Contracts Manager	Range: $75,000 – 80,000	.5
Accountant	Range: $75,000 – 80,000	.5

## Abbreviations

CIC: Community Initiated Care
HMHC: Healthy Minds Healthy Communities
ARPA: American Rescue Plan Act of 2021
LMHA: Local Mental Health Authority
MHPP: Mental Health Prevention and Promotion
CLC: Community Learning Circle
BLS: Bureau of Labor Statistics
ASK: AS + K Ask about Suicide to Save a Life Training
