# Correction: A microcosting approach for planning and implementing community‑based mental health prevention programs: what does it cost?

**DOI:** 10.1186/s13561-024-00524-4

**Published:** 2024-07-10

**Authors:** Sharmily Roy, Henry Shelton Brown, Lisa Sanger Blinn, Sarah Carter Narendorf, Jane E. Hamilton

**Affiliations:** 1 School of Public Health, University of Texas Health Science Center, 1200 Pressler St, Houston, Houston, TX 77030 USA; 2 The Harris Center for Mental Health and IDD, 9401 Southwest Freeway, Houston, TX 77074 USA; 3 Graduate College of Social Work, University of Houston, 3511 Cullen Blvd, Houston, TX 77204 USA; 4 Department of Psychiatry and Behavioral Sciences, McGovern Medical School, University of Texas Health Science Center, 1941 East Road, Houston, Houston, TX 77054 USA

**Correction:** ***Health Econ Rev*** **14, 35 (2024)**

10.1186/s13561-024-00510-w

Following publication of the original article [1], the authors identified the following errors in the affiliations:

1. Affiliation 1 (School of Public Health, Department of Management Policy and Community Health, University of Texas Health Science Center, Houston, 1200 Pressler St, Houston, TX 77030, USA) and affiliation 2 (School of Public Health, University of Texas Health Science Center, Houston, 1200 Pressler St, Houston, TX 77030, USA) are the same; thus, affiliation 1 can be deleted.
2. Sharmily Roy’s affiliations should be affiliations 1 and 4 instead of 1,2.

The original article [1] has been corrected.
