# Supplementary material for: A microcosting approach for planning and implementing community-based mental health prevention programs: what does it cost?
Source: Health Econ Rev. 2024 May 21;14:35. doi: 10.1186/s13561-024-00510-w (PMC11110374; doi:10.1186/s13561-024-00510-w)
Supplement: Supplementary file 1 — Supplementary Material 1. [file 13561_2024_510_MOESM1_ESM.docx]

**Appendix 2**

*Research and Internal Launch*

The implementing agency, Harris Center, invested a considerable amount of time in research and development in the quarter prior to the program's initiation and launch. While research costs are not directly related to the program implementation, these activities are described to inform future implementers who may have some of the same issues of initiating community facing programs in a traditionally clinical setting. Research and development time was spent in conceptualization of implementing the CIC model, proposal development and application for the ARPA funds available throughout the county, human resource planning, and legal support (Table). Since the LMHA, a clinical agency, had not previously implemented a community-based MHPP program, there was a sizable amount of time spent structuring management, legal, and administrative processes to allow community-based activities in the field. This area of effort by leadership and administrators is not in the program budget. This research and development time of these organizational leaders’ (i.e., vice president of community affairs, accounting, legal affairs) contribution to project development and planning activities was calculated to be about $86,733 to $90,930 through staff time, estimated to be about 1200 personnel hours. Program research, development and launch is described for informational purposes, but not included in microcosting analysis, since these may not be applicable for future implementers adapting the CIC model.

**Table:** HMHC Project Development Contribution by Harris Center Staff

| **Position** | **Annual Salary** | **Effort in Calendar Months** |
| --- | --- | --- |
| Executive Leadership | Range: 135,000 - 167,000 | 2 |
| Program Manager | Range: $70,000-90,000 | 2 |
| Senior Director | Range:  $90,000-120,000 | 1 |
| Government Affairs Director | Range:  $80,000-115,000 | 1 |
| Business Manager | Range: $70,000-90,000 | .5 |
| Contracts Manager | Range: $75,000-80,000 | .5 |
| Accountant | Range: $75,000-80,000 | .5 |
